# Analytical solution of a non-homogeneous boundary-value problem for the transport equation in an earth to air energy exchanger by initial base analysis method

**DOI:** 10.1016/j.mex.2022.101819

**Published:** 2022-08-12

**Authors:** Landry Jean Pierre Gomat, Diogène Vianney Pongui Ngoma

**Affiliations:** Laboratoire de Mécanique, Energétique et Ingénierie, Ecole Nationale Supérieure Polytechnique -ENSP-, Université Marien NGouabi, Brazzaville, BP 69, Congo

**Keywords:** Initial condition, Initial base analysis method, Analytical shape solution

## Abstract

In this paper, a one-dimensional time-dependent transport equation with inhomogeneous Dirichlet boundary conditions, often encountered in the study of an Earth to Air Energy Exchanger, is solved by a practical analytical method. The initial condition function is not randomly set to verify the transport equation at the initial time, but determined as a first approximation to the solution of the transport equation (at t=0). As in the homotopy analysis method, the solution of the transport equation is expressed by the set of a basis function constructed with the obtained initial condition function. The components of the solution in this base, are time’s functions deduced from the boundary conditions of the problem. Suitable endings are noticeable between the proposed model and experimental measurements.

## Highlights

• The approach consists in analytically determining the evolution of the properties or quantities of matter transported by the movement (here humid air) through the device (the Earth to Air Energy Exchanger).
• The method could be applied to solve many other practical convective transport problems.

## Graphical abstract

**Figure:**
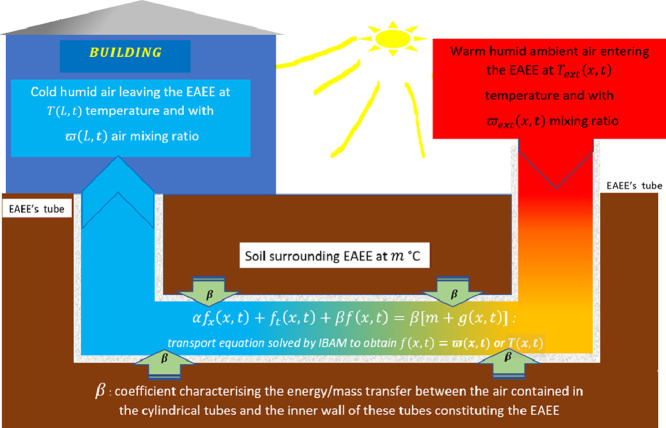

**Table:** Specifications table.

Subject area	Energy
More specific subject area:	Study of Earth to Air Energy Exchanger for thermal comfort in buildings and greenhouses.
Name of your method:	Initial Base Analysis Method
Name and reference of original method:	The Homotopy Analysis Method (HAM), ref:Yu C, Wang H, Fang D, Ma J, Cai X, Yu X, Semi-analytical solution to one-dimensional advective-dispersive-reactive transport equation using homotopy analysis method. j.hydrol. 2018; https://doi.org/10.1016/j.jhydrol.2018.08.041
Resource availability:	Gomat, Landry Jean Pierre (2022), “Experimental ambient air mixing ratio recorded at Strabourg 27.08.2016 by ICUBE”, Mendeley Data, V1, doi: 10.17632/66zbkd6rxn.1

## Method details

The Earth to Air Energy Exchanger (EAEE) is a quasi-passive ventilation device increasingly used for human comfort in buildings. It contributes to the reduction of energy consumption by man. It is a network of buried tubes that serves as an air duct from the outside to the inside of buildings to be cooled or heated [1], [2]. The tubes allow energy to be transmitted from the ground where they are buried to the air that circulates in them. Convective transport, diffusion and conduction phenomena are observed. The Fourier’s heat conduction and Fick’s diffusion functions are the perfect mathematical models in order to describe the transportation problems in various cases. According to the laws of conservation of mass and energy, Fick’s law diffusion functions ($\vec{j}=-\;D_{as}\vec{grad}\;\;\varpi \left(t\right)$, where ϖ(t) is the mixing ratio transported by the air flowing in an EAEE, and $D_{as}$ is a positive constant) and Fourier’s law heat conduction ($\vec{q}=-k\;\vec{grad}\;\;T\left(t\right)$, where T(t) is the temperature transported by air flowing in an EAEE and k is a positive constant), the transport problem thus posed can be represented by the one-dimensional convective equation in the form (1) below.

(1) $$\left\{ \begin{array}{c} \alpha \frac{\partial f(x,t)}{\partial x}+\frac{\partial f(x,t)}{\partial t}+\beta \;f\left(\eta ,t\right)=\beta \left[m+\;g\left(x,t\right)\right],\;\;\;\;\;\;\;\;0\leq x\leq L,\;\;\;\;t\geq 0\\ f\left(0,t\right)=f_{b}\left(t\right)\\ \lim_{x⟶\infty }f\left(x,t\right)=f\left(L,t\right)=m\\ f(x,0)=u(x) \end{array}\right.$$

where x represents the space between an arbitrary point and the source (length in semi-infinite space), t represents time, α represents the convection coefficient and β denotes the decay coefficient of f(x,t). All these coefficients and m are positive constants.

g(x,t) is a known function of the walls bounding the system and $f\left(x,t\right)\in C^{2}([0,\infty \left[\right)$ is the solution of model. The boundary conditions are of non-homogeneous Dirichlet types. $f_{b}\left(t\right)$ represents the variations of f(x,t) at the origin of space (at x=0). In many works encountered, simplifying assumptions are made to reduce the system (1) to a problem with homogeneous Dirichlet boundary conditions (g(x,t)=0) to solve it analytically. Otherwise, we proceed to a numerical solution (discretization by finite differences, finite elements or finite volumes,...) of the system (1). In all cases, the choice of the function u(x) indicating the initial condition of the problem is made randomly, making sure that it satisfies the transport equation of (1) at the initial time (at t=0). In this paper, an simple analytical method for solving the transport Eq. (1) of two physical quantities, air temperature and air mixing ratio in a Newtonian flow through an earth-air energy exchanger (EAEE), is presented. A one-dimensional study of the source-term transport equation in a semi-infinite spatial domain [3] is set up.

Rather than choosing the function u(x) at random, the method proposes to determine it as the solution of Eq. (1) [1] at the initial time (t=0).

The solution of problem (1) is then considered as an expansion in the basis of the function (1, u(x)) so that f(x,t) has components $\alpha_{1}\left(t\right)$ and $\alpha_{2}\left(t\right)$ in this basis. These components are uniquely a function of time and are determined through the boundary conditions of the problem.

The method has been implemented in two previous works [1], [2] which showed its relevance and the need to formalize the approach. In paper [1], the work consists of evaluating the dry air temperature at any time and any point in the cylindrical tubes of an EAEE. Article [2] is a study of the transport of humidity in the air circulating in the cylindrical tubes of an EAEE. The humidity was characterized in this study by the mixing ratio of water vapor and dry air.

The transport equation is encountered in the study of EAEEs which are natural ventilation devices transporting both temperature, humidity and other properties or matters contained in the air blown in their tubes - usually cylindrical - to be cooled, heated, humidified or dehumidified [4],...

EAEEs, like most energy exchangers, are helping to meet the world’s growing energy demand. The importance of an energy exchanger lies in its energy transfer coefficient which, when it improves the performance of the exchanger, makes it an important tool in solving energy consumption problems. Air conditioners (natural ventilation or not) or radiators, have required more and more, the improvement of energy transfer coefficients and even mass. Thus, EAEEs, whose tubes are the main exchange material, are associated with phase change materials (PCM), microfluids or nanofluids [5], [6].

As soon as the transport equation can be put in the form of problem (1), the analytical solution of this problem is easy by this method which can be called the initial basis analysis method (IBAM). The choice of the function u(x), the initial condition of the problem is subject to its correspondence with the physical reality of the initial conditions in the tubes of an EAEE. Determining it from the established equation and boundary conditions rather than choosing it at random is an objective and efficient approach. The initial condition must not only satisfy the transport equation, but also the boundary conditions of the problem at the initial time.

The initial condition representing a steady state before the uniform flow (x≤αt) of the fluid under study must verify the condition of steady state below:

(2) $$\frac{\partial f(x,0)}{\partial t}=\frac{\partial u(x)}{\partial t}=0,\;\;\;\;\;\;\;\;0\leq x\leq L$$

it comes:

(3) $$\alpha \frac{du(x)}{dx}+\beta \;u\left(x\right)=\beta \left[m+\;g\left(x,0\right)\right]$$

with boundary conditions given by Eqs. (4) and (5) below:

(4) $$u\left(0\right)=f_{b}\left(0\right)$$

(5) $$\;\lim_{x⟶\infty }u\left(x\right)=u\left(L\right)=m,\;\;\;\;\;\lim_{x⟶\infty }g\left(x,0\right)=0$$

From where one obtains the Eq. (6) below giving the initial condition of the problem in the considered device (here the EAEE):

(6) $$u(x)=m+\left[\;f_{b}\left(0\right)-m\;+A\left(x\right)-A\left(0\right)\right]\;exp\left(-\frac{\beta }{\alpha }x\right),\;\;\;\;\;\;\;\;0\leq x\leq L$$

with

(7) $$A\left(x\right)=\frac{\beta }{\alpha }\int \;g\left(x,0\right)\;exp\left(\frac{\beta }{\alpha }x\right)dx$$

As in Homotopy Analysis Method (HAM) [7], it is assumed that all solution f(x,t) of Eq. (1) can be expressed by the set of a base function (1,f(x,0)) in the form (8) in which $\alpha_{1}\left(t\right)$ and $\alpha_{2}\left(t\right)$ are time dependent functions yet to be determined by taking into account the boundary conditions of the problem, it is verified that function (8) is effectiveness solution of Eq. (1).

(8) $$f\left(x,t\right)=\alpha_{1}\left(t\right)+\alpha_{2}\left(t\right)\;f\left(x,0\right)$$

Solution (8) matches the boundary conditions in the problem (1) such that:

(9) $$\alpha_{1}\left(t\right)+\alpha_{2}\left(t\right)\;f_{b}\left(0\right)=f_{b}\left(t\right)$$

(10) $$\alpha_{1}\left(t\right)+\alpha_{2}\left(t\right)\;m=m$$

The function $f_{b}\left(t\right)$ is a known function with explicite analytical expression.

It is obtained the analytical solution (11) of the transport Eq. (1):

(11) $$f\left(x,t\right)\;=\;m\;\;\frac{f_{b}\left(t\right)-f_{b}\left(0\right)}{m-f_{b}\left(0\right)}\;\;+\;u(x)\;\frac{m-f_{b}\left(t\right)}{m-f_{b}\left(0\right)}$$

or better in the most contracted form (12):

(12) $$f\left(x,t\right)\;=\;m\;+\left[f_{b}\left(t\right)-m\right]\;exp\left(-\frac{\beta }{\alpha }x\right)+\Gamma \left(x,t\right)$$

The function Γ(x,t) bespeaks the damping of the additional fluctuations resulting from the boundaries of the system and modeled by the function g(x,t). It is given by:

(13) $$\Gamma \left(x,t\right)=\left[A(x)-A(0)\right]\;\;\frac{m-f_{b}\left(t\right)}{m-f_{b}\left(0\right)}\;exp\left(-\frac{\beta }{\alpha }x\right),\;\;\;\;0\leq t<\infty ,\;\;0\leq x\leq \delta$$

As soon as one can put the transport equation of the problem in study in the form of the Eq. (1), one is insured to obtain the analytical solution (12) of the problem. What matters is to identify the coefficients and the functions so as to present them as in the diffusion Eq. (1).

## Applications and discussion

In this subsection, the application of the method for the transportation of dry air temperature and wet air mixing ratio by an EAEE is implemented. The subsection presents and discusses the numerical results and the comparison with the experimental data. The main focus is to discuss the trend in the data/results and the possible effects or underlying physical significance of these trends on the performance of these applications. The reliability of the method depends on the three parameters α,β and m:

• the air flow velocity α in the tubes of the EAEE influences its impregnation on the internal walls of the tubes to improve exchanges.
• The frequency β which depends on the energy or mass transfer coefficient in addition to the velocity α, indicates the performance of the EAEE in terms of energy or mass exchanges.
• The coefficient m which is determined by the average temperature $T_{msol}$ of the area where the EAEE is buried.

While the choice of the air flow velocity α in the EAEE tubes is easy, a point of honor must be placed on the calculation of β and m. For an EAEE, the parameter m depends on the area of its burial in the soil. It is strongly correlated with the temperature of the area under consideration. Models for estimating the average soil temperature have been the subject of several studies such as [8].^1^ β is a rather difficult parameter to evaluate. It reflects the energy exchange efficiency of the device because it carries the energy or mass transfer coefficient by convection. The latter is the subject of much improvement work. Bae and Rohsenow [9], Shah [10], Camaraza-Medina et al. [11] and Rifert’s work [12], [13]) on the determination of the energy and mass transfer coefficient finds good values of β. The possible effects of the presented results on the performance of such applications should be discussed. With the above in mind, the following applications were calculated. Since the parameters β and m can be improved, their effects on the expected results are evaluated.

### Transportation of the temperature by an EAEE

As in the work [1], the study of the transportation of the temperature T(x,t) of dry air by an EAEE with cylindrical tube has led to the following transport equation of initial condition:

(14) $$\alpha \frac{dT(x,0)}{dx}+\beta \;T\left(x,0\right)=\beta \;m,\;\;\;\;\;\;\;\;0\leq x\leq L$$

where α represents the uniform velocity of the air flow, β is the energy transfer frequency throughout the inner surface of a cylindrical tube, m is the average soil temperature where the tube is buried, L is the length of the whole exchanger and

(15) $$T\left(0,t\right)=T_{ext}\left(t\right),\;\;\;\;\;\;\;\;\;\;T\left(L,t\right)=m$$

The continuum expression of $T_{ext}\left(t\right)$ - the ambient air temperature - is given by the equation (16) below, which is the Newton’s polynomial approximation of the data recorded in August 27, 2016 in Strasbourg from the time 00h00 to 23h00.

(16) $$\begin{array}{ccc} T_{ext}\left(t\right) & = & 21.48-0.71t+0.115(t-1)t-0.007881317(t-2)(t-1)t+\\ & & -\;0.003011783(t-3)(t-2)(t-1)t+0.001700818(t-6)(t-3)(t-2)(t-1)t+\\ & & -\;0.000329223*(t-9)(t-6)(t-3)(t-2)(t-1)t+\\ & & +\;3.75403\times 10^{-5}\left(t-12\right)\left(t-9\right)\left(t-6\right)\left(t-3\right)\left(t-2\right)\left(t-1\right)t+\\ & & -\;3.02561\times 10^{-6}\left(t-15\right)\left(t-12\right)\left(t-9\right)\left(t-6\right)\left(t-3\right)\left(t-2\right)\left(t-1\right)t+\\ & & +\;1.90943\times 10^{-7}\left(t-18\right)\left(t-15\right)\left(t-12\right)\left(t-9\right)\left(t-6\right)\left(t-3\right)\left(t-2\right)\left(t-1\right)t+\\ & & -\;1.1498\times 10^{-8}\left(t-21\right)\left(t-18\right)\left(t-15\right)\left(t-12\right)\left(t-9\right)\left(t-6\right)\left(t-3\right)\left(t-2\right)\left(t-1\right)t+\\ & & +\;7.30757\times 10^{-10}\left(t-22\right)\left(t-21\right)\left(t-18\right)\left(t-15\right)\left(t-12\right)\left(t-9\right)\left(t-6\right)\left(t-3\right)\left(t-2\right)\left(t-1\right)t+\\ & & -\;5.77514\times 10^{-11}\left(t-23\right)\left(t-22\right)\left(t-21\right)\left(t-18\right)\left(t-15\right)\left(t-12\right)\left(t-9\right)\left(t-6\right)\left(t-3\right)\left(t-2\right)\left(t-1\right)t; \end{array}$$

Following the method, it comes:

(17) $$T\left(x,0\right)=m+\left[T_{ext}\left(0\right)-m\right]\;exp\left(-\frac{\beta }{\alpha }x\right),\;\;\;\;\;\;\;\;0\leq x\leq L$$

and

(18) $$T\left(x,t\right)=m+\left[T_{ext}\left(t\right)-m\right]\;exp\left(-\frac{\beta }{\alpha }x\right)$$

**Fig. 1:**
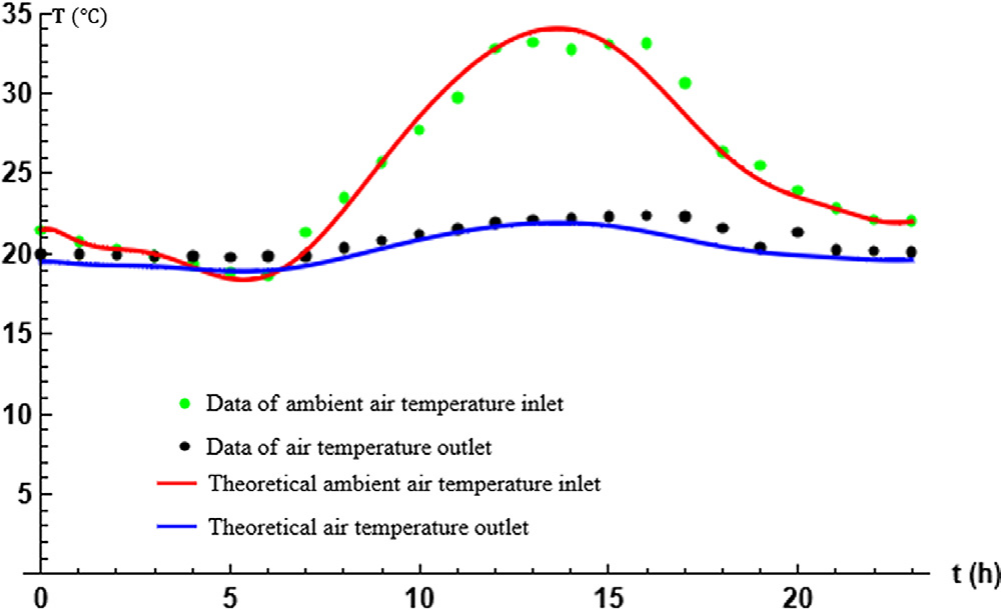
Comparison between analytical and experimental results.

Figure 1 shows the comparison between the theoretical results obtained and the data recorded by the temperature variation curves between the inlet and outlet of the EAEE. The experimentally imposed flow velocity α of the air in the cylindrical tubes of the EAEE is $0.51\;\text{m}\;\text{s}{}^{-1}$ for a total length of the exchanger tubes of L=17.50 m. Comparison of the analytical results with the experimental results, performed graphically (as shown in Fig. 1), shows very good agreement between these results.

#### Effect of the air overall energy transfer frequency β in the tubes

The parameter β represents the overall energy transfer frequency of the air in the tubes. The parameter β is varied, by setting m, to evaluate its effect on the theoretical results obtained and the performance of the exchanger. Table 1 gives the values used.

**Table 1:** Table of values of β in transport Eq. (14).

β (s${}^{-1}$)	0.048	0.060	0.081

**Fig. 2:**
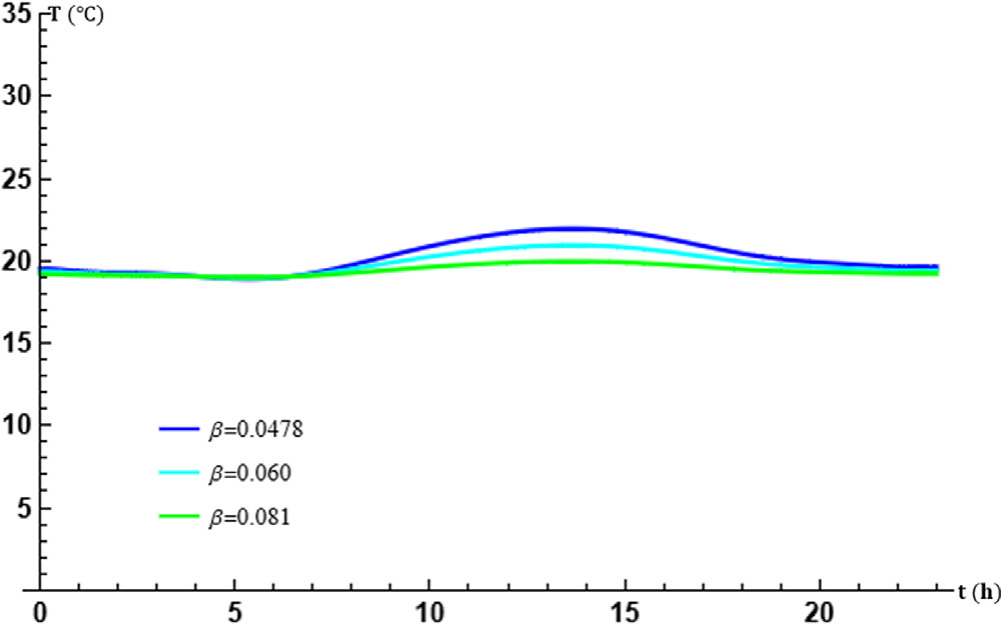
Effect of the air overall energy transfer frequency β in the tubes at x=L.

**Fig. 3:**
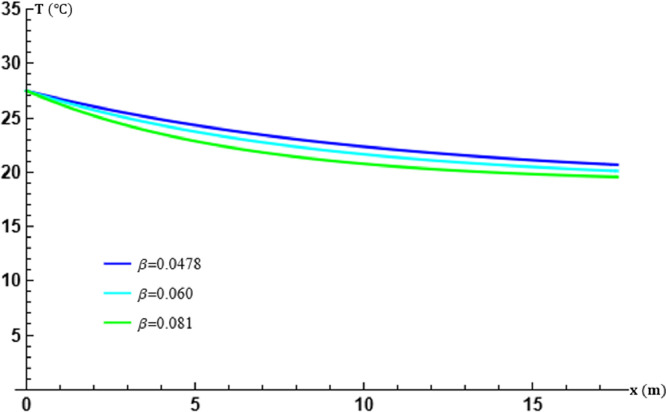
Effect of the air overall energy transfer frequency β in the tubes at t=17:30.

The graphs in Figs. 2 and 3 show that the calculated temperature variations are in perfect agreement with those experimentally recorded. The variations in β do not change the appearance of the temperature curve at the outlet of the EAEE, but clearly indicate significant changes in the efficiency of the exchanger. The higher β, the better the energy exchange in the cylindrical tubes of the EAEE. The agreement of these temperature values with the experimental results depends on the calculation of β at fixed m. This shows that if β is determined incorrectly, the theoretical results could be strongly affected even if the method of calculating the transfort equation is very good. From its accuracy follows the conformity of the theoretical results with the experimental results.

#### Effect of the average temperature m of the soil surrounding tubes

The parameter m represents the average temperature of the soil surrounding the exchanger tubes. Its value is estimated, most often by considering the soil as a continuous material without any source of matter or energy, semi-infinite, in which the energy stored at the surface of the soil is damped. This phenomenon reflects the thermal inertia of the subsoil, which then constitutes an inexhaustible source of energy [1], [8]. The parameter m is varied at a fixed β in order to evaluate these effects on the theoretical results obtained and the results of the study. Effects on the theoretical results obtained and on the performance of the exchanger. Table 2 gives the values used.

**Table 2:** Table of values of m in transport Eq. (14).

m (${}^{\circ }$C)	17.31	19.02	21.30

**Fig. 4:**
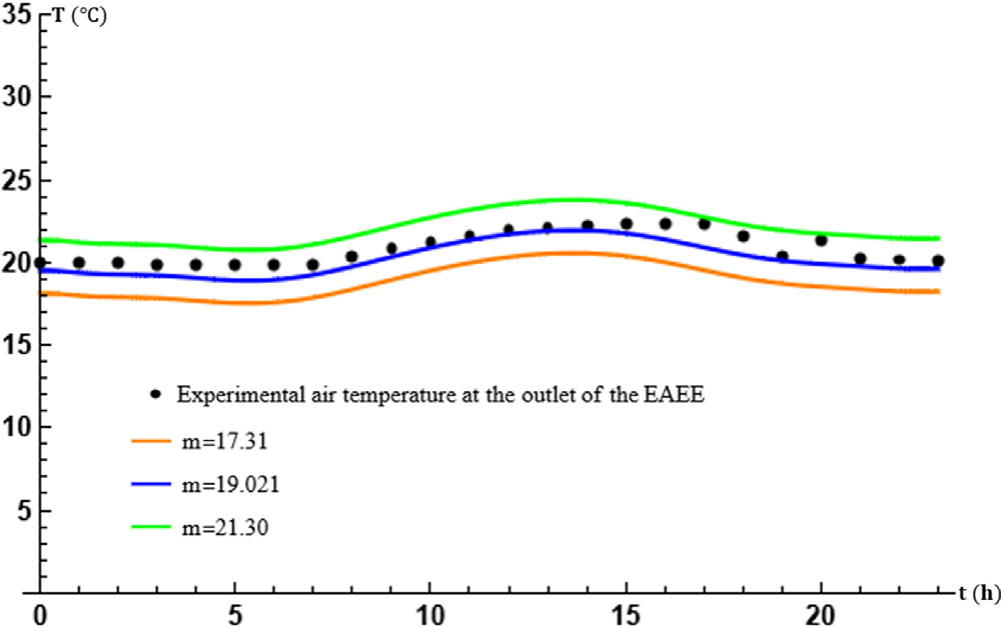
Effect of the average temperature m of the soil surrounding tubes at x=L.

**Fig. 5:**
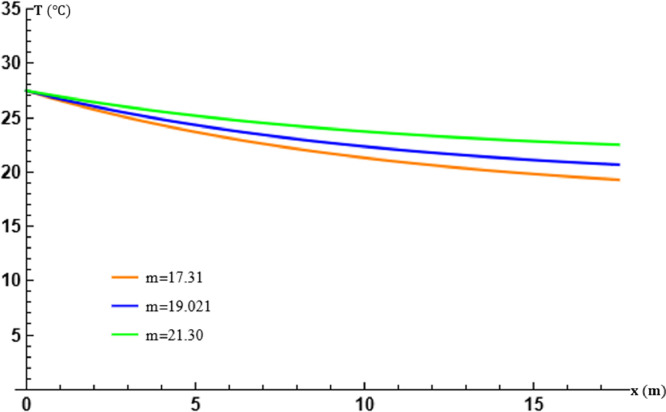
Effect of the average temperature m of the soil surrounding tubes at at t=17:30.

The plots in Figs. 4 and 5 show, as for the effects of β, that the calculated temperature variations are in perfect agreement with those experimentally recorded, but the agreement of the values of these temperatures depends on the accuracy of the calculation of m at fixed β. A poor estimate of m would therefore lead to discrepancies between the theoretical and experimental results, even when the method of calculating the transport equation is very good.

### Transport of mixing ratio by an EAEE

As in the work [2], the study of the transportation of the mixing ratio of moist air has led to the following transport equation:

(19) $$\alpha \frac{d\varpi (x,0)}{dx}+\beta \;\varpi \left(x,0\right)=\beta \;\left[m_{\varpi }+g_{\varpi }\left(x,0\right)\right],\;\;\;\;\;\;\;\;0\leq x\leq L$$

where $g_{\varpi }\left(x,0\right)$ is deduced from the transport equation as a shape form:

(20) $$g_{\varpi }\left(x,0\right)=p\;exp\left(-qx\right)$$

α is the uniform velocity of the air flow, β represents the mass transfer frequency throughout the inner surface of a cylindrical tube, $m_{\varpi }$ is the air mixing ratio at the average temperature of the soil where the tube is buried, L is the length of the whole exchanger.

Boundary conditions being as below:

(21) $$\varpi \left(x=0,t\right)=\varpi_{ext}\left(t\right)$$

(22) $$\varpi \left(x=L,t\right)=m_{\varpi }$$

The analytical expression of $\varpi_{ext}\left(t\right)$ is given by:

(23) $$\begin{array}{ccc} \varpi_{ext}\left(t\right) & = & 0.015869364-0.000698304t+0.000123056\left(t-1\right)t-1.02156\times 10^{-5}\left(t-2\right)\left(t-1\right)t+\\ & & -\;1.90368\times 10^{-5}\left(t-3\right)\left(t-2\right)\left(t-1\right)t+1.25922\times 10^{-5}\left(t-4\right)\left(t-3\right)\left(t-2\right)\left(t-1\right)t+\\ & & -\;2.45843\times 10^{-6}\left(t-5\right)\left(t-4\right)\left(t-3\right)\left(t-2\right)\left(t-1\right)t+\\ & & +\;2.708\times 10^{-7}\left(t-8\right)\left(t-5\right)\left(t-4\right)\left(t-3\right)\left(t-2\right)\left(t-1\right)t+\\ & & -\;1.96232\times 10^{-8}\left(t-11\right)\left(t-8\right)\left(t-5\right)\left(t-4\right)\left(t-3\right)\left(t-2\right)\left(t-1\right)t+\\ & & +\;9.54239\times 10^{-10}\left(t-14\right)\left(t-11\right)\left(t-8\right)\left(t-5\right)\left(t-4\right)\left(t-3\right)\left(t-2\right)*\left(t-1\right)t+\\ & & -\;2.52675\times 10^{-11}\left(t-17\right)\left(t-14\right)\left(t-11\right)\left(t-8\right)\left(t-5\right)\left(t-4\right)\left(t-3\right)\left(t-2\right)\left(t-1\right)t+\\ & & -\;8.87049\times 10^{-13}\left(t-20\right)\left(t-17\right)\left(t-14\right)\left(t-11\right)\left(t-8\right)\left(t-5\right)\left(t-4\right)\left(t-3\right)\left(t-2\right)\left(t-1\right)t+\\ & & +\;2.14557\times 10^{-13}\left(t-21\right)\left(t-20\right)\left(t-17\right)\left(t-14\right)\left(t-11\right)\left(t-8\right)\left(t-5\right)\left(t-4\right)\left(t-3\right)\left(t-2\right)\left(t-1\right)t+\\ & & -\;2.31568\times 10^{-14}\left(t-22\right)\left(t-21\right)\left(t-20\right)\left(t-17\right)\left(t-14\right)\left(t-11\right)\left(t-8\right)\left(t-5\right)\left(t-4\right)\left(t-3\right)\times \\ & & \times \left(t-2\right)\left(t-1\right)t+2.00642\times 10^{-15}\left(t-23\right)\left(t-22\right)\left(t-21\right)\left(t-20\right)\left(t-17\right)\left(t-14\right)\left(t-11\right)\left(t-8\right)\times \\ & & \times \left(t-5\right)\left(t-4\right)\left(t-3\right)\left(t-2\right)\left(t-1\right)t-1.55843\times 10^{-16}\left(t-24\right)\left(t-23\right)\left(t-22\right)\left(t-21\right)\left(t-20\right)\times \\ & & \times (t-17)(t-14)(t-11)(t-8)(t-5)(t-4)(t-3)(t-2)(t-1)t; \end{array}$$

The analytic form (23) calls out the Newton’s polynomial interpolation from the data of Strasbourg dated August 27th between 00h00 and 23h00.

Following the method, it comes for the air mixing ratio initial condition:

(24) $$\varpi \left(x,0\right)=m_{\varpi }+\left[\;\varpi_{ext}\left(0\right)-m_{\varpi }\;+A_{\varpi }\left(x\right)-A_{\varpi }\left(0\right)\right]\;exp\left(-\frac{\beta }{\alpha }x\right)$$

with

(25) $$A_{\varpi }\left(x\right)=\;\frac{\beta }{\alpha }\;\;\int g_{\varpi }\left(x,0\right)\;exp\left(\frac{\beta }{\alpha }x\right)dx,\;\;\;\;\;\;\;\;0\leq x\leq L$$

For the air mixing ratio evolution in the tube of the EAEE the Eq. (26) below:

(26) $$\varpi \left(x,t\right)\;=\;m_{\varpi }\;+\left[\varpi_{ext}\left(t\right)-m_{\varpi }\right]\;exp\left(-\frac{\beta }{\alpha }x\right)+\Gamma_{\varpi }\left(x,t\right)$$

(27) $$\Gamma_{\varpi }\left(x,t\right)=\left[A_{\varpi }\left(x\right)-A_{\varpi }\left(0\right)\right]\;\;\frac{m_{\varpi }-\varpi_{ext}\left(t\right)}{m_{\varpi }-\varpi_{ext}\left(0\right)}\;exp\left(-\frac{\beta }{\alpha }x\right),\;\;\;\;\;0\leq x\leq L$$

**Fig. 6:**
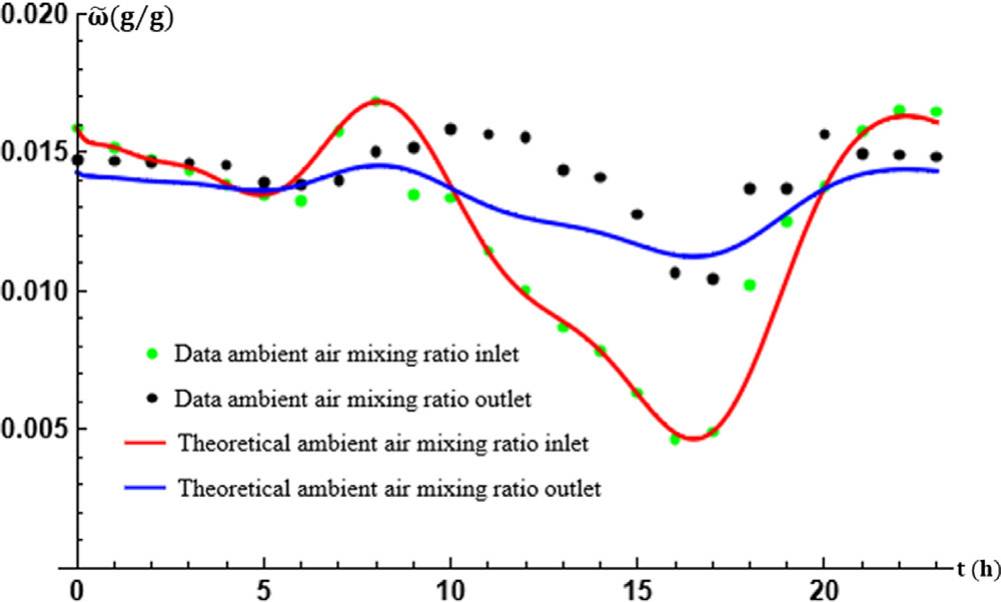
An analytical approximation of experimental air mixing ratio recorded.

Figure 6 shows the comparison between the theoretical results obtained and the data recorded by the mixing ratio variation curves between the inlet and outlet of the EAEE. The experimentally imposed flow velocity is $\beta =0.51\;\text{m}\;\text{s}{}^{-1}$ for a total exchanger tube length of L=17.50 m. Comparison of the analytical results with the experimental results performed graphically (as shown in Fig. 6) shows very good agreement between these results.

#### Effect of the air overall mass/energy transfer frequency in the tubes

The overall mass/energy transfer frequency of the air in the tubes is represented by the parameter β. The parameter β is varied by setting $m_{\varpi }$ to appreciate its effects on the theoretical results obtained and the performance of the exchanger with p=0.0225 and q=3.2001. This gives the values in Table 3 below.

**Table 3:** Table of values of β in transport Eq. (19).

β (s${}^{-1}$)	0.01688	0.04688	0.08688

**Fig. 7:**
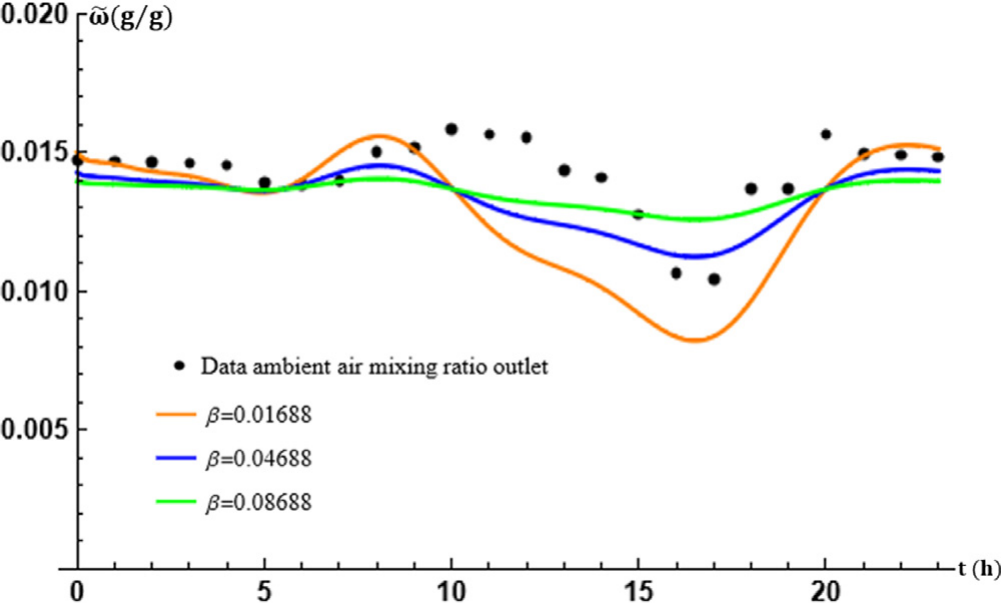
Effect of the air overall mass/energy transfer frequency β in the tubes at x=L.

**Fig. 8:**
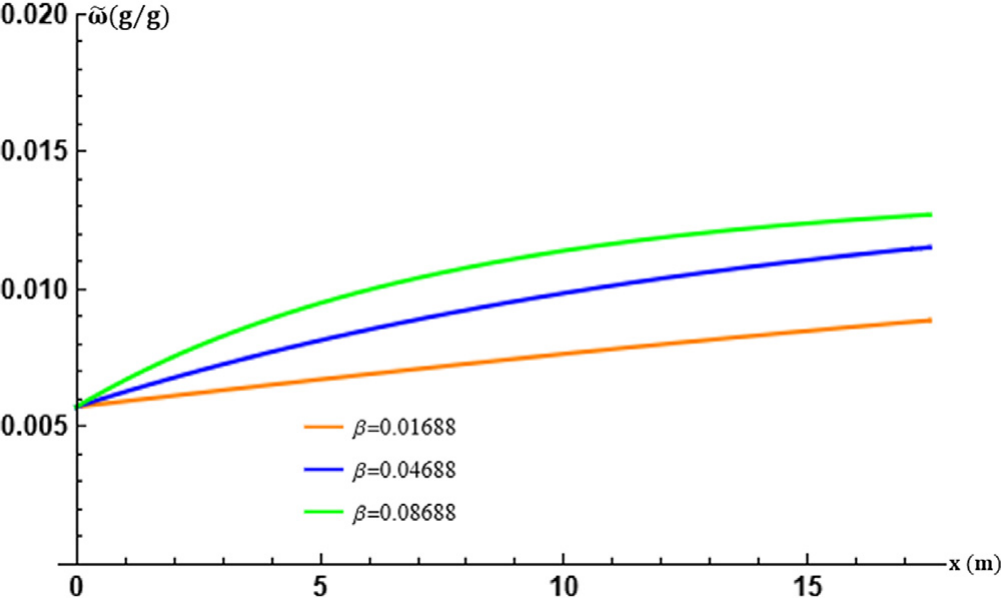
Effect of the air overall mass/energy transfer frequency β in the tubes at t=17:30.

The graphs in Figs. 7 and 8 thus show that the variations in the mixing ratio of moist ambient air in the EAEE tubes are in perfect agreement with those experimentally recorded despite the shift in values which, as for temperature, depend on the calculation of β at fixed $m_{\varpi }$. The determination of β is crucial to obtain theoretical results in agreement with the experimental ones.

#### Effect of the average temperature of the soil surrounding tubes

The mixing ratio of the saturated moist air flowing through the EAEE tubes is given, at the average ground temperature m, by the parameter $m_{\varpi }$. By fixing β and varying $m_{\varpi }$, its effects on the theoretical results obtained are evaluated. The variations of $m_{\varpi }$ induce the variations of the coefficient p contained in the source term of Eq. (19). This results in the values retained in the following Table 4.

**Table 4:** Table of values of $m_{\varpi }$ and p in transport Eqs. (19) and (20).

$m_{\varpi }$ (g g${}^{-1}$)	0.0123	0.0136	0.0143
p (g g${}^{-1}$)	0.4$\times 10^{-7}$	3.4$\times 10^{-7}$	3.4$\times 10^{-7}$

**Fig. 9:**
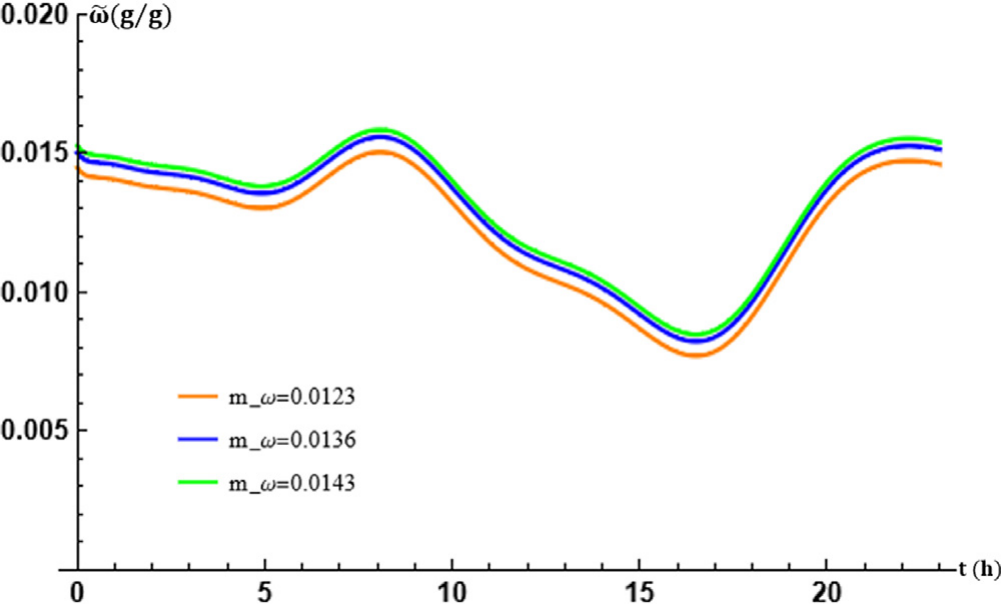
Effect of the air overall mass/energy transfer frequency in the tubes at x=L.

**Fig. 10:**
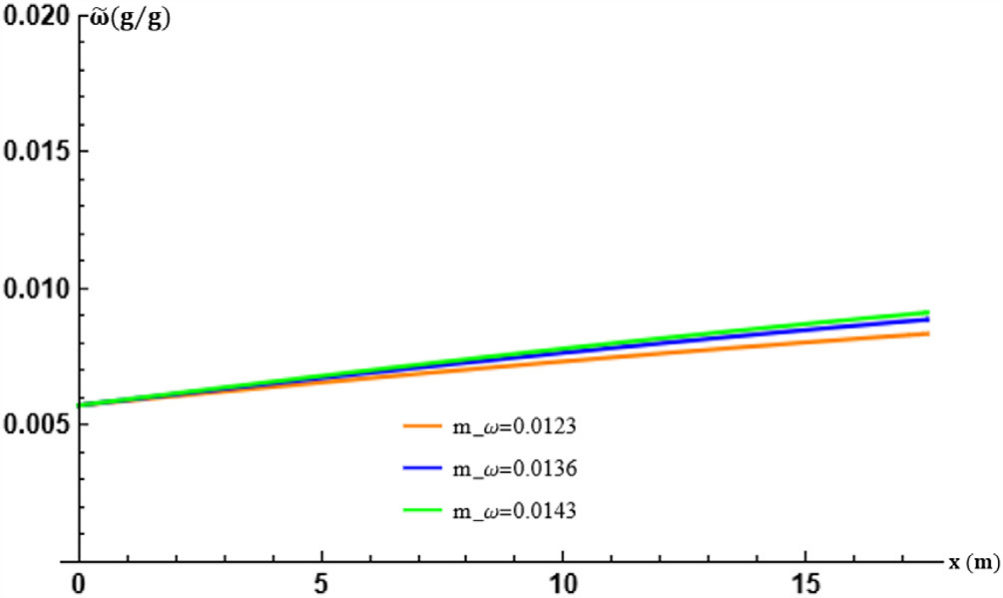
Effect of the air overall mass/energy transfer frequency in the tubes at t=17:30.

The graphs of Figs. 9 and 10 show, as for the parameters already studied, the effects of the $m_{\varpi }$ coefficient on the solution of (1). The same conclusion can be done.

## Conclusion

It is found that the solution of the method is in good agreement with the experimental solutions, which demonstrates the reliability and efficiency of the method, despite the fact that the analytical expressions of the temperature and the mixing ratio of the ambient air were constructed from Newton’s interpolations to within 10% for temperature and to within 20% for mixing ratio. Indeed, the study of the effects of the parameters influencing the obtained results indicates that the resolution method allows to obtain exceptionally efficient results provided that the parameters determining the physical phenomena are properly calculated. The average temperature of the soil in which the EAEE is located could just as easily be $m=17.31{\;}^{\circ }$C, $m=19.02{\;}^{\circ }$C as $m=21.30{\;}^{\circ }$C, but these differences do not concern the method of solving the transport equation. The same applies to the calculation of β. Most of the work on EAEEs, as on many other devices that take advantage of energy transfers, indicates that the calculation of energy transfer coefficients is crucial in energy transfer calculations in general. Physical (through the use of PCMs, microfluids, or nanofluids) and theoretical (through the accuracy of the calculation to obtain energy transfer coefficients) improvement of energy transfer coefficients remains the keystone of calculations in this area. This work allows the analytical obtaining, always more reliable than numerical, of solutions of the transport equation in the EAEE. One certainly wants to believe that the application of this method can be used to solve many other practical convective transport problems. In perspective, it would be a question of seeing if the equation of energy transport in the vortex tubes could be put in the form (1) at least in the main cylinders of these tubes [14].

## CRediT authorship contribution statement

**Landry Jean Pierre Gomat:** Conceptualization, Methodology, Validation, Writing – review & editing. **Diogène Vianney Pongui Ngoma:** Investigation, Writing – review & editing.

## Declaration of Competing Interest

The authors declare that they have no known competing financial interests or personal relationships that could have appeared to influence the work reported in this paper.

## Footnotes

1 The calculation of the energy transfer coefficient by convection is presented in the coefficient β. This one is subject to improvement because it is still not obvious to determine. Apart from that, the improvement of the energy exchange in the exchanger can be obtained by associating new material in the constitution of the EAEE. This is where PCMs and nanofluids come in as suggested by the Reviewer5.
